# A Novel Feature Selection Approach Based on Tree Models for Evaluating the Punching Shear Capacity of Steel Fiber-Reinforced Concrete Flat Slabs

**DOI:** 10.3390/ma13173902

**Published:** 2020-09-03

**Authors:** Shasha Lu, Mohammadreza Koopialipoor, Panagiotis G. Asteris, Maziyar Bahri, Danial Jahed Armaghani

**Affiliations:** 1 Civil Engineering College, Liaoning Technical University, Fuxin 123000, China; lilyherb@163.com; 2 Faculty of Civil and Environmental Engineering, Amirkabir University of Technology, Tehran 15914, Iran; Mr.koopialipoor@aut.ac.ir; 3 Computational Mechanics Laboratory, School of Pedagogical and Technological Education, 14121 Heraklion, Greece; 4 Department of Building Structures and Geotechnical Engineering, Higher Technical School of Architecture, Universidad de Sevilla, 41012 Sevilla, Spain; mazbah@alum.us.es; 5 Institute of Research and Development, Duy Tan University, Da Nang 550000, Vietnam

**Keywords:** fiber-reinforced concrete, punching shear capacity, tree model, feature selection, hybrid predictive models

## Abstract

When designing flat slabs made of steel fiber-reinforced concrete (SFRC), it is very important to predict their punching shear capacity accurately. The use of machine learning seems to be a great way to improve the accuracy of empirical equations currently used in this field. Accordingly, this study utilized tree predictive models (i.e., random forest (RF), random tree (RT), and classification and regression trees (CART)) as well as a novel feature selection (FS) technique to introduce a new model capable of estimating the punching shear capacity of the SFRC flat slabs. Furthermore, to automatically create the structure of the predictive models, the current study employed a sequential algorithm of the FS model. In order to perform the training stage for the proposed models, a dataset consisting of 140 samples with six influential components (i.e., the depth of the slab, the effective depth of the slab, the length of the column, the compressive strength of the concrete, the reinforcement ratio, and the fiber volume) were collected from the relevant literature. Afterward, the sequential FS models were trained and verified using the above-mentioned database. To evaluate the accuracy of the proposed models for both testing and training datasets, various statistical indices, including the coefficient of determination (R^2^) and root mean square error (RMSE), were utilized. The results obtained from the experiments indicated that the FS-RT model outperformed FS-RF and FS-CART models in terms of prediction accuracy. The range of R^2^ and RMSE values were obtained as 0.9476–0.9831 and 14.4965–24.9310, respectively; in this regard, the FS-RT hybrid technique demonstrated the best performance. It was concluded that the three hybrid techniques proposed in this paper, i.e., FS-RT, FS-RF, and FS-CART, could be applied to predicting SFRC flat slabs.

## 1. Introduction

Various projects in the field of civil engineering, e.g., residential buildings, office blocks, and parking stations, utilize reinforced concrete flat slabs due to the fact that the structure produced by the two-way cast-in-place concrete slabs will be able to offer a cost-effective structural system for engineers as well as architects [1,2]. Various features of the reinforced concrete flat slabs, including flat soffit, can remarkably facilitate the installation of rebar as well as formwork [3]. Moreover, these structures can result in a reduced overall height of the story. The benefits of reinforced concrete flat slabs have attracted the attention of a large number of researchers working on the reactions of such structures in both theoretical and experimental studies [4,5,6]. According to the available literature, the punching shear capacity of the slab-column connections can be considered as the maximum strength of a reinforced concrete flat slab [1]. On the other hand, compared to the punching load, the residual strength of a slab after punching is significantly lower. Therefore, after punching the slab at one of the columns, the neighboring columns can rapidly become overloaded and develop a failure state upon punching. This can result in the escalating breakdown of those buildings where flat slab components are used [1]. There are many building collapses reported in the literature triggered by failure on punching, resulting in deaths as well as significant economic loss. As an example, Schousboe [7] reported a case where a 24-story building collapsed during construction in 1973 in Virginia. Once investigations were completed on the incident, it was reported that the collapse was caused by a shear failure in the slab component utilized on one of the top floors. In addition, King and Delatte [8] discussed the breakdown of a building complex with 16 stories in the US because of too low punching shear strength of the flat slab component. To prevent such cases of collapse, a number of recent studies have focused on the failure mechanism of such structures. They have attempted to improve the punching shear capacity of slabs using empirical equations. On the other hand, there has been an increase in the popularity of steel fibers in the field of structural engineering [9]; thus, these fibers have been used as reinforcement in concrete flat slabs as a means to improve their punching shear capacity [10,11,12]. It is also worth mentioning that a number of experimental studies (e.g., [3]) have confirmed that the punching shear capacity can be improved by reinforcing concrete flat slabs using steel fibers. This has resulted in the widespread application of steel fiber-reinforced concrete (SFRC) flat slabs to various construction building-related projects. However, an important issue about the slab-column connection is the fact that the design codes have been established for such structures, currently (e.g., the ACI 318-11 standard [13]). Therefore, it is necessary to modify the current codes so that they can adapt to the design process for the SFRC slabs. In this regard, Narayanan and Darwish [14] proposed an equation based on the compressive zone’s strength over inclined cracks, the pull-out shear forces exerted upon the steel fibers in the direction of such cracks, and the shear forces reinforced by membrane actions as a means to determine the punching shear capacity of the SFRC. Moreover, Harajli et al. [15] proposed a design equation based on linear regression, which can be used for analyzing the contribution of the concrete and fibers to the total punching shear strength. On the other hand, Choi et al. [16] presented a theoretical study evaluating the effectiveness of a design equation, which is supported by the assumption related to the response of tensile reinforcement before the occurrence of punching shear failure. Additionally, Maya et al. [3] attempted to evaluate three different prediction equations applied to calculate the punching shear capacity by acquiring empirical data available in the literature. In another relevant study, Gouveia et al. [17] analyzed an experimental investigation focusing on the behavior of the SFRC up to failure. More recently, Gouveia et al. [18] carried out another experimental study aimed at evaluating the punching shear capacity of the SFRC slab-column connections. Moreover, Gouveia et al. [19,20] implemented experimental studies to assess the load capacity of the SFRC flat slabs subjected to vertical loads incremented in a monotonic manner as well as reversed horizontal cyclic loading. Another study conducted by Kueres et al. [21] investigated the response of reinforced concrete flat slabs as well as column bases to the punching shear force by employing the fracture kinematics of the slabs. Kueres and Hegger [22] suggested a kinematic theory based on two different parameters for the punching shear in reinforced concrete slabs that lack shear reinforcement. A new experimental approach was proposed by Einpaul et al. [23] to record the creation and progress of cracks in punching test samples. Simões et al. [24] analyzed the measurements for the kinematics as well as the crack development, corresponding to punching failures. The results obtained from this analysis were then utilized for establishing a mechanical model, aiming at better understanding the punching shear failures. A review of available literature showed that the prediction process for the shear punching capacity of SFRC is often concentrated on using modified design equations and simple statistical methods. While the theoretical prediction models are highly important for assessing the relation between the shear punching capacity of the SFRC and the factors affecting it. It should be noted that punching shear behavior is a very complex phenomenon, necessitating the evaluation of other approximation and estimation methods. During the last decades, the lack of adequate and reliable empirical or analytical relations for the evaluation of the shear punching capacity of flat slabs has resulted in attracting the interest of researchers dealing with non-deterministic techniques.

In the light of the above discussion, over the last decades, the applications of artificial intelligent (AI) and machine learning (ML) techniques have rapidly grown, and new intelligent models have been developed to solve problems in science and engineering (especially civil engineering) [25,26,27,28,29,30,31,32,33,34,35,36,37,38,39,40,41,42,43,44,45,46,47,48,49,50,51,52,53,54,55,56,57,58,59,60,61]. Hoang [62] studied the sophisticated data analysis approach for estimating shear punching capacity. Accordingly, PLMR (piecewise linear multiple regression) as well as ANN (artificial neural network) were chosen in their research as predictive techniques, and then the shear punching capacity of SFRC flat slabs was predicted. Furthermore, by training the PLMR model, an automatic sequential approach was utilized. Hoang’s study was focused on the PLMR ML approach since its configuration can clearly be explained, depicted, and understood. The PLMR was identified as an appropriate instrument for modeling shear punching capacity. On the other hand, the ANN model was used by Armaghani et al. [31] to determine the shear capacity of concrete beams. By examining laboratory samples and comparing them with classical models, they introduced ANN as a model of high flexibility. In another research conducted by Asteris et al. [36], various structures of the ANN model were developed. They presented the network weights as a new procedure of modeling. Given that the performance of the AI models can be enhanced, Asteris et al. [63] improved an ANN structure using the normalization technique for predicting the mechanical properties of sandcrete materials. The results showed that the developed model offered a high performance compared to experimental models. Due to the development of intelligent models, the capabilities of optimization algorithms were also considered. Sun et al. [29] proposed an artificial bee colony algorithm to optimize the developed models. In their study, different concrete samples were optimized and compared with real samples. Although AI techniques are able to solve many problems related to science and engineering, they can introduce a new model, which is black-box, and its configuration cannot easily be explained and understood by researchers and engineers [64,65,66,67,68,69,70,71,72]. On the other hand, tree-based models can offer broader capabilities in the field of nonlinear problem-solving. By developing tree-based models, a tree model can be extracted, which is easy to understand and apply [34,73,74]. One of the weaknesses in recent research simulating the intelligent models is the lack of control over important data for simulation. These data can be better achieved if it can be managed in the best way, and the simulation process can be optimized by selecting superior features.

In this research, to determine the prediction model with the best performance, the tree-based models were compared to each other. The data used in different studies have various properties, each of which can change the simulation process. Because recent research focuses less on such data, this paper implemented a new process by selecting important features from the data. This process ultimately helped intelligent models to increase model performance. In order to train and test the above-mentioned ML models, a dataset consisting of 140 experimental data specimens was acquired from the available literature. This dataset included six explanatory variables, i.e., the depth of the slab, the effective depth of the slab, the length of the column, the compressive strength of the concrete, the reinforcement ratio, and the fiber volume. These variables were used to forecast the punching shear capacity of the SFRC flat slabs. The rest of the current paper is organized as follows: Section 2 deals with the formulation of the study. Section 3 discusses the iterative process performed to identify the structure of the tree-based models. Section 4 presents the results of the developed models. Finally, Section 5 concludes the whole study.

## 2. Formulation

### 2.1. Prediction Equations to Calculate the Punching Shear Strength of SFRC Slabs

It is possible to estimate the punching shear strength of SFRC using the mechanical model of the CSCT (i.e., the critical shear crack theory). In the following, the punching shear strength of SFRC with and without transverse reinforcement has been discussed [75,76]. The punching shear strength for reinforced concrete slabs that do not have transverse reinforcement can be expressed as [76]:

(1) $$V_{R,C}=\frac{3}{4}b_{0}d\sqrt{f_{c}}\frac{1}{\left(1+15\frac{\psi d}{d_{g0}+d_{g}}\right)}$$

where Ψ signifies the maximal rotation of the slab, d denotes the effective depth of the slab, $b_{o}$ signifies the control perimeter (which is considered at a distance of d/2 from the face of the column), $f_{c}$ denotes the compressive strength of the concrete, $d_{g}$ signifies the aggregate size, and $d_{g0}$ is the reference aggregate size that can be set to 16 mm. Along the surface of the failure, which is determined by the critical shear crack, the overall shear strength achieves the contributions from the concrete and the steel fibers [77]. The following equation can be utilized for calculating the punching shear strength:

(2) $$V_{R}=V_{R,C}+V_{R,f}$$

where $V_{R,C}$ represents the contribution from the concrete, and $V_{R,\;f}$ signifies the contribution from the fibers. Moreover, Voo and Foster [78] presented a formulation for quantifying the tensile strength generated by the fibers over a plane with unit area. This equation can be expressed as:

(3) $$\sigma_{tf}=K_{f}\cdot \alpha_{f}\cdot \rho_{f}\cdot \tau_{b}$$

where $K_{f}$ signifies the global orientation factor, $q_{f}$ denotes the volume of the fiber, $s_{b}$ is the bond stress between the fibers and the concrete mix, and $a_{f}$ represents the aspect ratio parameter for the steel fibers. Based on this equation, the overall punching shear contribution from the fibers can be computed as follows [3]:

(4) $$V_{R,f}=\int_{A_{P}}^{}\sigma_{tf}\left(\psi ,\xi \right)dA_{P}$$

where ξ signifies the distance between a point and the soffit of the slab, and $A_{p}$ represents the horizontally projected area of the punching shear failure surface. It is worth mentioning that the integration in this equation makes it possible to reach a closed-form solution in order to calculate the contribution from the fiber [77]. Moreover, according to the notion of the average bridging stress as well as the kinematic assumption [77], it is possible to calculate the contribution from the fiber using the equation presented below [3]:

(5) $$V_{R,f}=A_{P}\sigma_{tf}\left(\frac{\psi d}{6}\right)$$

Using the above equation, Maya et al. [3] proposed a simplified equation for calculating the contribution from the concrete. Their equation is presented as follows:

(6) $$V_{R,C}=\frac{2b_{0}d\sqrt{f_{C}}}{3\gamma_{c}}\frac{1}{1+20\frac{\psi d}{d_{g0}+d_{g}}}$$

In this equation, $\gamma_{c}$ represents the partial safety factor for the concrete, which is equal to 1.5. 

### 2.2. Experimental Data Collection

**Figure 1:**
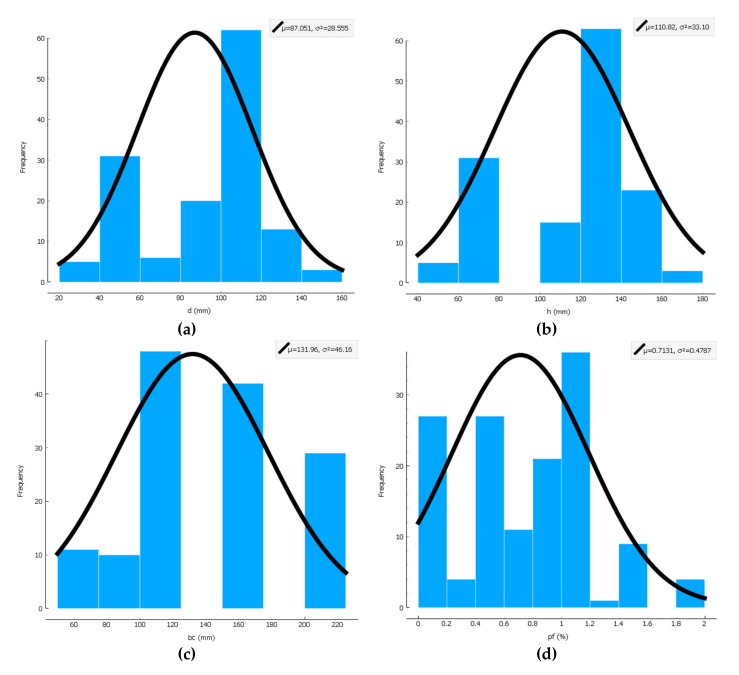

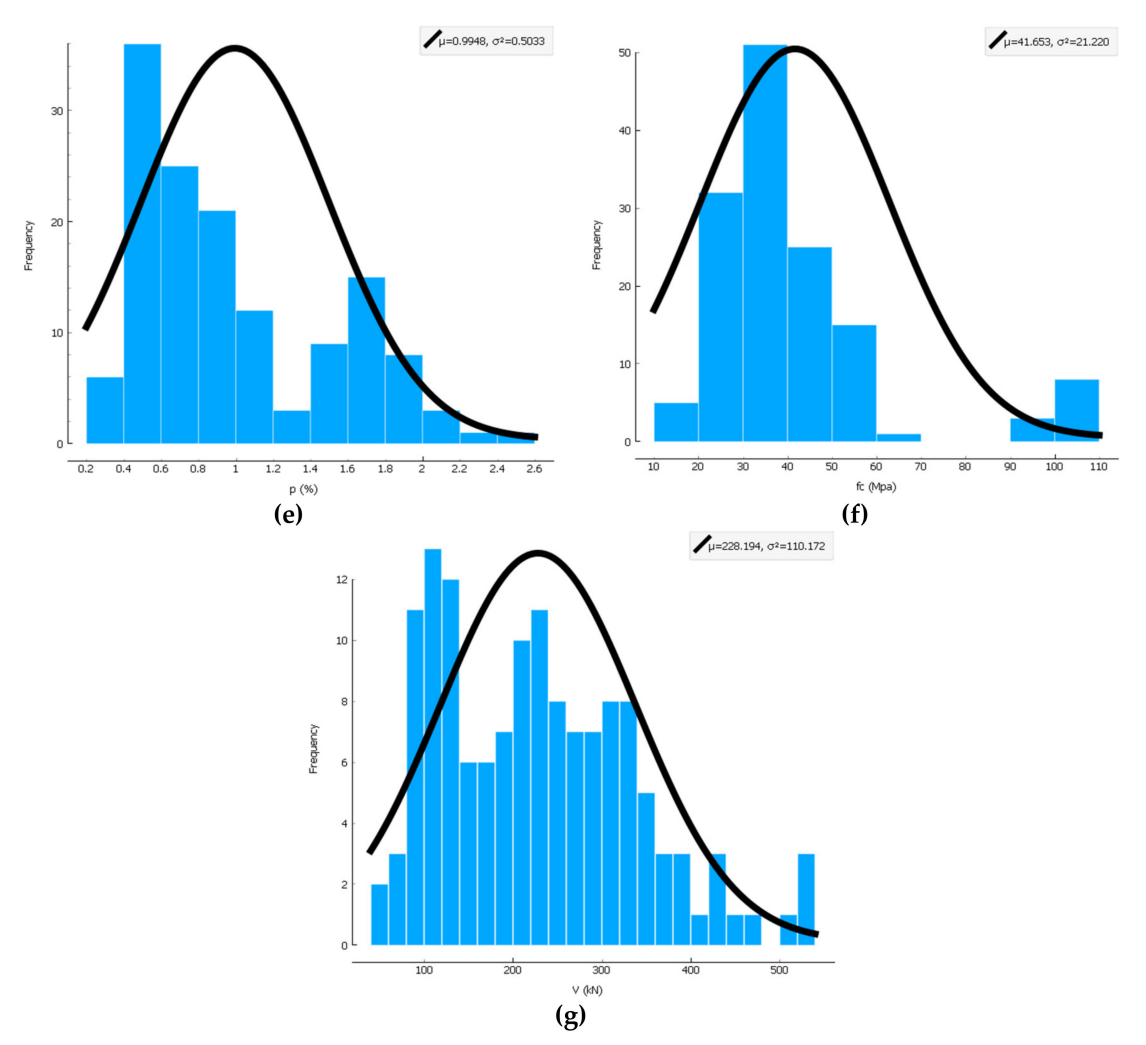
Histogram chart for all data, including (**a**) the effective depth of the slab, (**b**) the depth of the slab, (**c**) the length of the column, (**d**) the fiber volume, (**e**) the reinforcement ratio, (**f**) the compressive strength of the concrete, (**g**) the punching shear strength.

A dataset consisting of 140 test specimens as well as six factors governing the punching capacity, i.e., the depth of the slab (*h*), the effective depth of the slab (*d*), the length of the column (*bc*), the compressive strength of the concrete (*f_c_*), the reinforcement ratio (ρ), and the fiber volume ($\rho_{f}$), was collected for training and testing the ML models. The main influential parameters for the punching capacity samples included geometry dimensions and materials used. The geometry of the samples used in this research included the range of *h =* 55–180 mm, *d* = 39–150 mm, *bc* = 60–225. The data points in this dataset were acquired from the experimental studies available in the literature [12,14,15,79,80,81,82,83,84,85,86,87,88,89,90,91]. Table 1 presents the statistical descriptions of the variables in this dataset to predict the punching shear strength (V). Moreover, Figure 1 depicts the histograms for the output and the input parameters. As shown in Figure 1, the two parameters μ and $\sigma^{2}$ represent the mean of data and variance of the data, respectively. Data quality can be determined using the normal distribution implemented for input and output data. Note that the data applied to predictive models should be selected with high accuracy.

## 3. Methodology

### 3.1. Random Forest

In 2001, Breiman [92] developed the random forest (RF) model that is a non-parametric ensemble classifier based on the flexible decision tree algorithm. This approach is, in fact, an expansion over the classification and regression tree, consisting of hybridization of numerous trees where bootstrap samples are employed to generate each individual tree [93,94]. In this approach, the algorithm employed for constructing the model automatically selects random parts of the training data. Moreover, in the training process, each branch of the tree at each node will be determined by a randomized subset of the variables. Furthermore, each individual tree is expanded in order to minimize the classification error; however, the result is affected by the random selection. The main objective of RF is to determine to what extent the prediction error increments as the data output for specific variables is permutated. Therefore, this approach is able to identify the significance of each variable when all variables are controlled [95,96]. 

### 3.2. Classification and Regression Trees

The classification and regression trees (CART) approach is a non-parametric regression method that is among the most widely-used ML approaches [93]. This approach is highly flexible since it can utilize any type of numeric and binary data, while the result is not affected by the monotone transformations and various measurement scales [97]. To construct the decision trees in CART, a binary partitioning algorithm is often used [98]. Furthermore, in order to deal with missing data in a specific factor, regression trees are usually utilized through the replacement process [93]. In this approach, in order to prevent the overfitting of terminal nodes in the tree, the splits are recursively snipped [93]. The CART method applies the equation below to classification problems; this method is based on comparing the distribution of the target attribute with two child nodes:

(7) $$I\left(split\right)={\left[0.5{\left(e|\left(1-e\right)\right)}^{u}\underset{k}{\sum }\left|P_{L}\left(K\right)-P_{R}\left(K\right)\right|\right]}^{2}$$

where k signifies the target classes, $PL\left(k\right)$ is the probability distribution target on the left side of the nodes, $PR\left(k\right)$ is the probability distribution target on the right side of the nodes, and u is the penalty on the splits [99]. 

### 3.3. Random Tree 

The random tree (RT) approach is a supervised classification model, which was first proposed by Breiman [92]. Similar to RF, RT is based on ensemble learning. In the RT approach, there are a number of learners who operate on their own. The notion of bagging is applied to constructing a decision tree, and it provides a randomly selected set of samples. The main difference between a standard tree and RF involves the splitting of the node. In RF, this splitting is performed based on the best predictor among a subset of predictors; however, the standard tree utilizes the elite split among all variables. RT is an ensemble of three predictors, i.e., forests, and it can deal with regression as well as classification applications. When the RT algorithm is executed, the tree classifier receives the input data. All the available trees classify the inputs. Finally, the class with the highest frequency will be output by the system. Since the training error is computed internally, cross-validation or bootstraps are not required for estimating the accuracy of the training stage. It is worth mentioning that the output for the regression problems is calculated by taking the average of the responses of all the forest members [100]. Moreover, the error for this approach is calculated based on the ratio of misclassified vectors to all the vectors present in the original dataset. 

## 4. Simulation and Discussion

**Figure 2:**
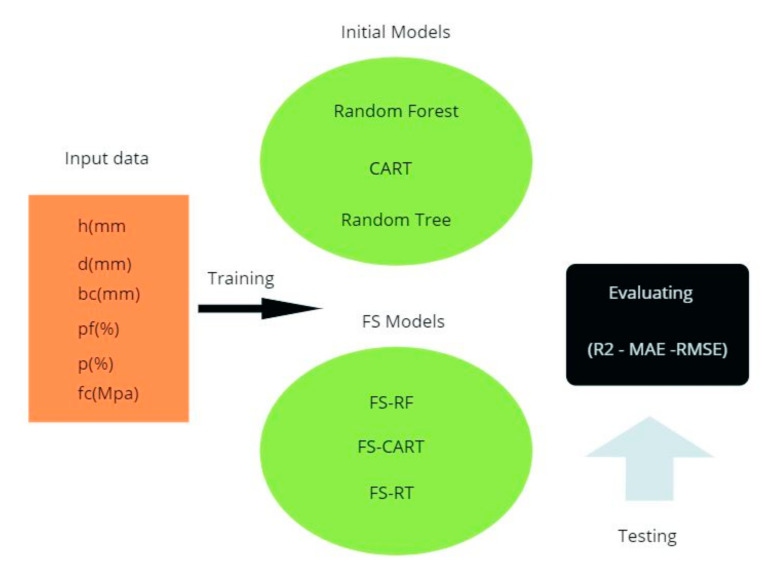
The methodology process of this research.

This section compares the performance of the proposed tree models with the performance of the feature selection (FS) hybrid models. It should be noted that a repetitive random subsampling, consisting of 20 training and testing runs, was carried out in order to assess the prediction performance of the model in a reliable manner. For each of the runs, 70 percent of the available data was employed as the training data for the model, estimating the punching shear strength, while the remaining 30 percent of the data was employed to test the model [101,102,103]. In addition, along with the *RMSE* (root mean square error), the *MAPE* (mean absolute percentage error), the *MAE* (mean absolute error), and the *R^2^* (coefficient of determination) were used to assess the performance of the model [104,105,106]. These performance evaluation indices are calculated using the following equations:

(8) $$MAPE=\frac{100\%}{N_{d}}\overset{N_{d}}{\underset{t=1}{\sum }}\frac{Y_{A,i}-Y_{P,i}}{Y_{A,i}}$$

(9) $$MAE=\frac{1}{N_{d}}\overset{N_{d}}{\underset{t=1}{\sum }}\left|Y_{A,i}-Y_{P,i}\right|$$

(10) $$R^{2}=\frac{SS_{yy}-SSE}{SS_{yy}}$$

where $Y_{A,\;i}$ indicates the actual value of the punching shear capacity of the i−th instance, while $Y_{P,\;i}$ represents the predicted value for the same parameter, and $N_{d}$ signifies the number of data instances in the selected dataset. To compute the value of *R^2^*, the values for $SS_{yy}$ and SSE must be calculated. These are obtained using the following equations:

(11) $$SS_{yy}=\overset{N}{\underset{i=1}{\sum }}{\left(Y_{A,i}-Y_{A,m}\right)}^{2}$$

(12) $$SSE=\overset{N}{\underset{i=1}{\sum }}{\left(Y_{A,i}-Y_{P,i}\right)}^{2}$$

where $Y_{A,\;m}$ signifies the mean of the actual punching shear capacity. Figure 2 presents the methodology process of this research in order to solve the problem.

### 4.1. RF

**Figure 3:**
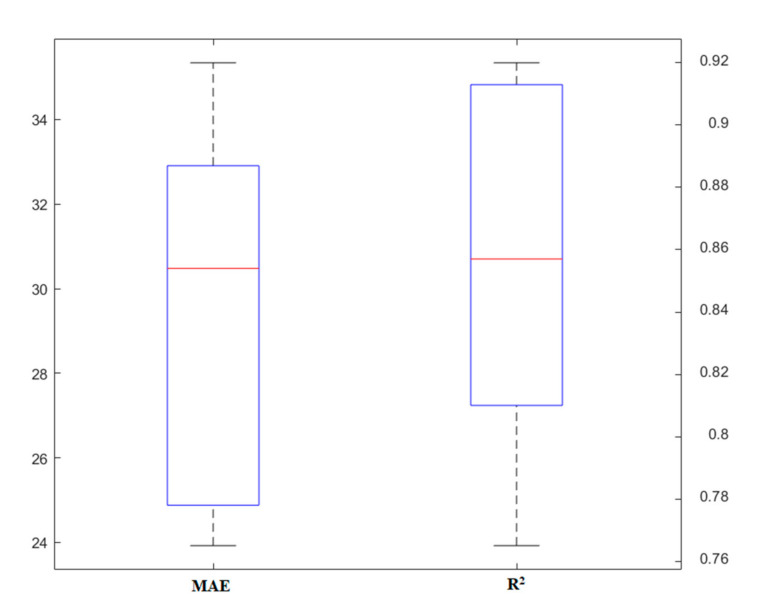
The results of the random forest (RF) model based on a different number of trees.

The simulation of different methods requires an examination of the main parameters to control the quality of the results. In this part, the modeling process of the RF technique is described. Since this model is based on the use of a tree, its important and effective parameters are similar to the base model. Initially, the data collected from the literature were divided into two parts: training and testing. Proper segmentation affects the simulation results, the flexibility of the models, and their accuracy. Due to the fact that in the simulations, about 70–80% of the data is recommended to be used for training purposes, in this study, 70% of the data was allocated to this part. Two parameters, i.e., tree number and tree depth, were identified as the two main keys to the RF model. Various models were implemented to design the appropriate structure for the prediction of the punching shear capacity of SFRC flat slabs so that the best conditions could be evaluated. Figure 3 shows the variations of models made based on the number of trees. To compare the performance of models, in this section, two statistical indices, *MAE* and *R^2^,* were used. The number of trees for this problem ranged from 1 to 10. As shown in this figure, the model changes generally provide an accuracy of *R^2^* = 0.82–0.9. This indicates that the parameter of tree number has a deep impact on model performance. Furthermore, *MAE* changes confirm this and show that the *MAE* rate decreases as model accuracy increases.

**Figure 4:**
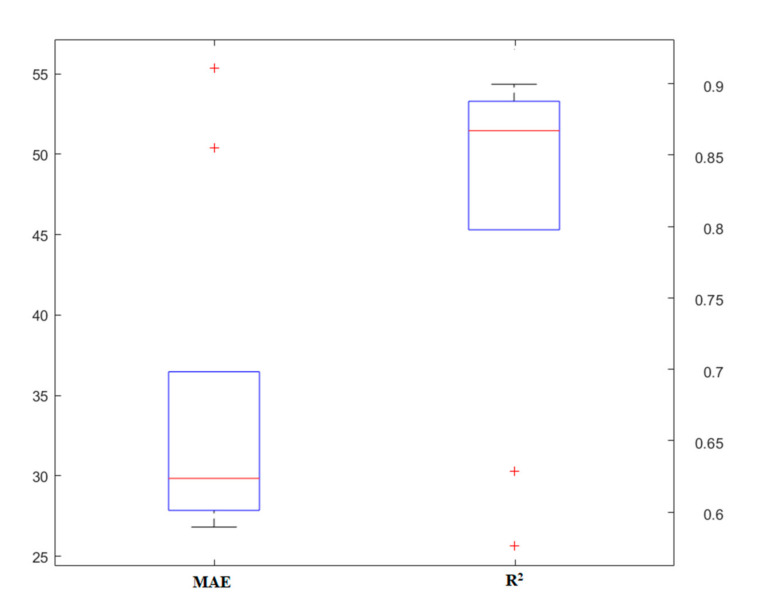
The results of the RF model based on the depth of the tree.

**Figure 5:**
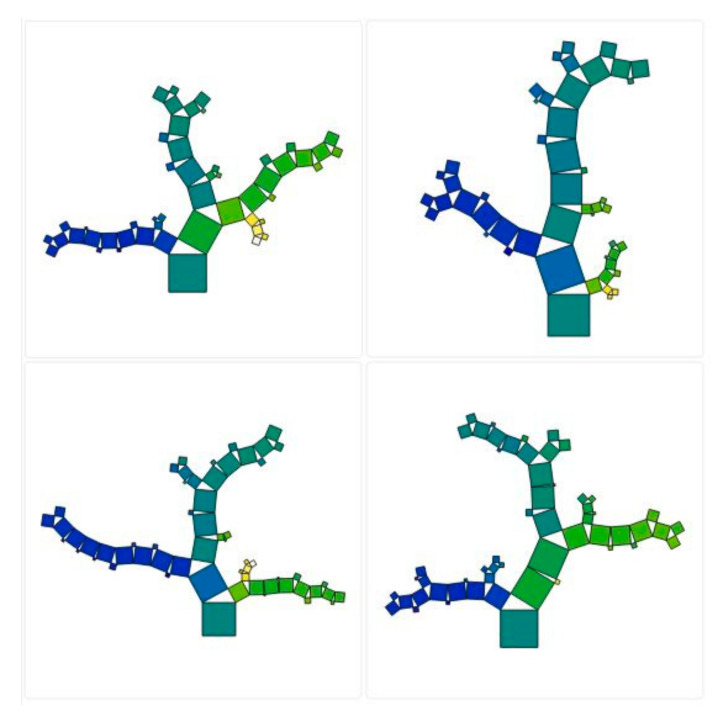
The structure of the RF model in predicting the punching shear capacity of steel fiber-reinforced concrete (SFRC) flat slabs.

Although the main part of the RF model design can be achieved with the number of trees, the tree depth parameter also has an important effect on the structure of the proposed model. The depth of the tree actually allows each tree to grow to a specific size. As shown in Figure 4, the range of changes in tree depth is smaller, and the closer to depth (10), the smaller the range of change. Figure 5 shows one of the suitable structures for this model. According to this figure, the number of trees and their depth can be well achieved by how they are grown.

### 4.2. CART

**Figure 6:**
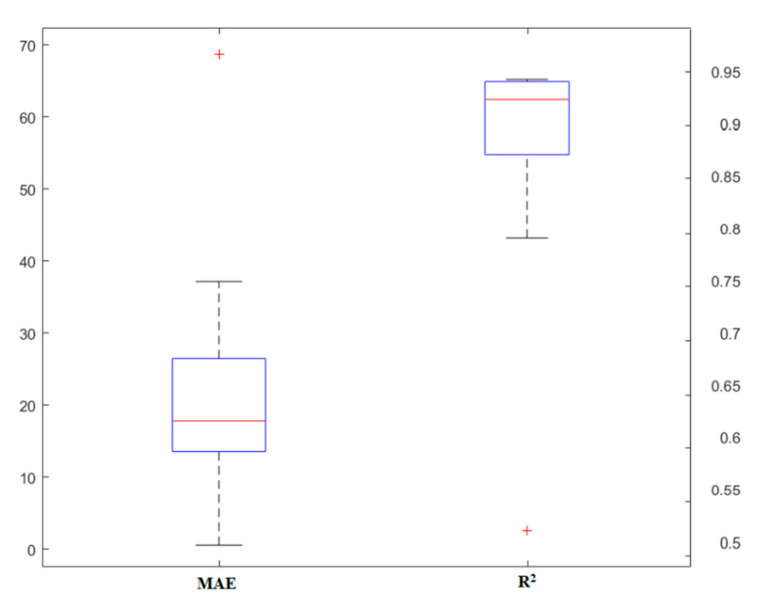
The result of the classification and regression trees (CART) model, based on the tree depth.

This model was used as one of the tree models in this study for the prediction of the punching shear capacity of SFRC flat slabs. Due to the close similarities to the tree structure, different cases of this model are also affected by this basic model. Ninety-eight data (70% of the total data) were allocated for designing and training the CART model. The number of inputs of this model (set to six) is in accordance with Table 1. The evaluation of the punching shear capacity of the SFRC flat slabs, with different methods, can be introduced as an alternative solution. The CART model attempted to obtain the best model through sensitivity analysis. Due to the same conditions with tree models, tree depth was evaluated as the most effective parameter. Figure 6 shows the effect of different changes in the models made. At the same time, it can be understood that the CART model offers fewer changes than the RF model and much higher accuracy. This suggests that it can be used as a superior model in predicting the punching shear capacity of SFRC flat slabs. The accuracy of this model can be achieved up to about *R^2^* = 0.95, with changes to tree depth.

### 4.3. RT

**Figure 7:**
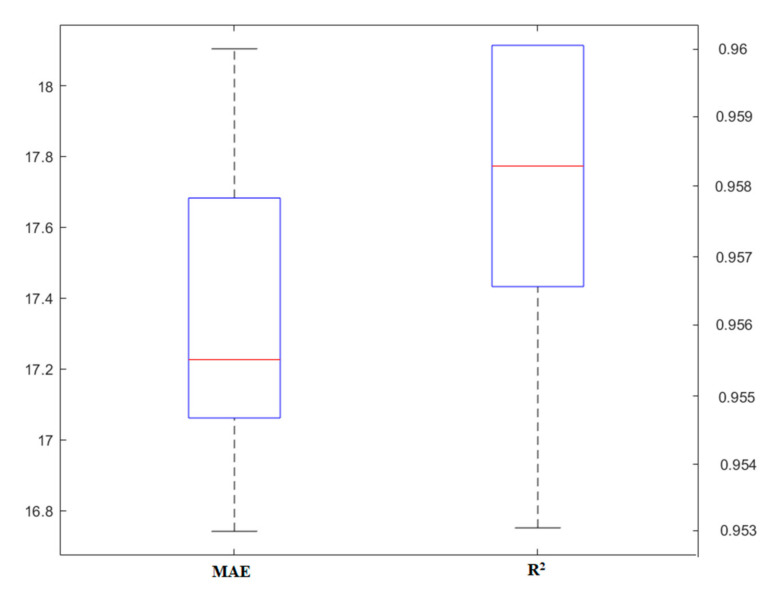
The results of the random tree (RT) model, based on the number of models.

**Figure 8:**
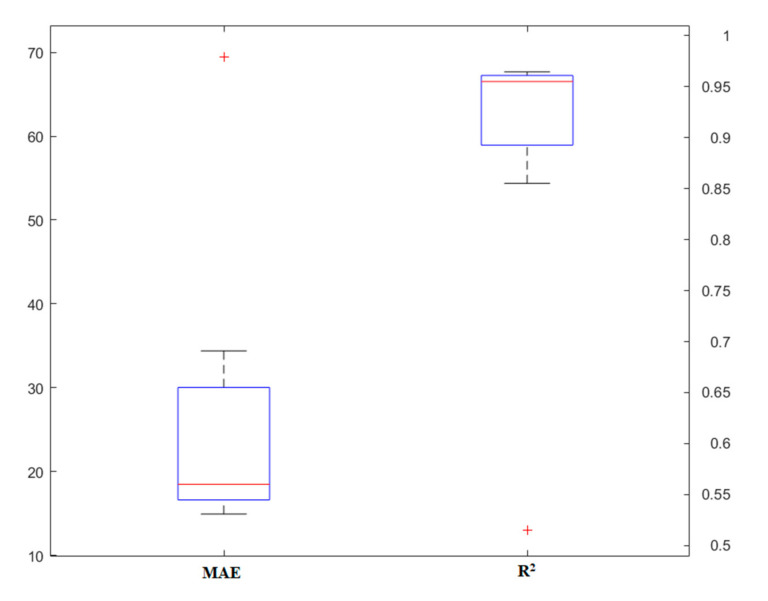
The results of the RT model, based on the tree depth.

Finally, the third model was implemented to predict the punching shear capacity of the SFRC flat slabs. This model also has different parameters, the most important of which are the number of models and the depth of the tree for each model. Initially, different models were designed so that the effect of each parameter could be well-identified. Figure 7 and Figure 8 show the results of the number of models and the depth of the tree, respectively. In general, from these two diagrams, it can be concluded that unlike the previous models, here, compared to the depth of the tree, the number of models has a greater impact on the simulation. In addition, it can be noted that this model offers a higher capability than the RF model, and the results are in a more acceptable range. Under these conditions, this model can be introduced as one of the highly-accurate predictive methods applicable to estimating the punching shear capacity of SFRC flat slabs.

### 4.4. FS Hybrids

In the previous sections, basic models were implemented to simulate and evaluate the punching shear capacity of SFRC flat slabs. The effects of their various parameters were examined, and finally, with more knowledge, the power of each model could be obtained. Because the models were trained without considering any changes in the data, they were used as baseline models for this research. In various simulations, the number of input parameters and data quality have significant effects on the results. Therefore, it is important to be focused on selecting outstanding and effective features, and then we can come up with high prediction performance models. This research implemented a process for designing an appropriate FS model. The process is such that the base models in the previous section were selected as comparative models, and then models based on them used the FS conditions. This process continues until it reaches a stable state and higher accuracy. The FSs used in this computational loop include various ranges of statistical tests to determine the superior features of the data. These include different statistical distributions, different dimensional of computational, weighting parameters, data outlier criteria, etc. Finally, hybrid models designed on this basis were tested to assess their flexibility. The results of the base and hybrid models are given in Table 2. As can be seen in this table, the hybrid models offer higher quality and accuracy in both training and testing modes. FS-RF, FS-CART, and FS-RT models obtained an accuracy of *R^2^* = 0.9476, 0.9608, and 0.9831 for training data, respectively. The same models provided accuracy of *R^2^* = 0.9190, 0.9454, and 0.9581 for testing data, respectively. These results show that they are more powerful models compared to the base models. By examining the errors of the models, it can be concluded that the errors have been reduced to an acceptable level compared to the initial models (i.e., single models). Therefore, in this study, the hybrid models are found capable of predicting the punching shear capacity of SFRC flat slabs.

**Figure 9:**
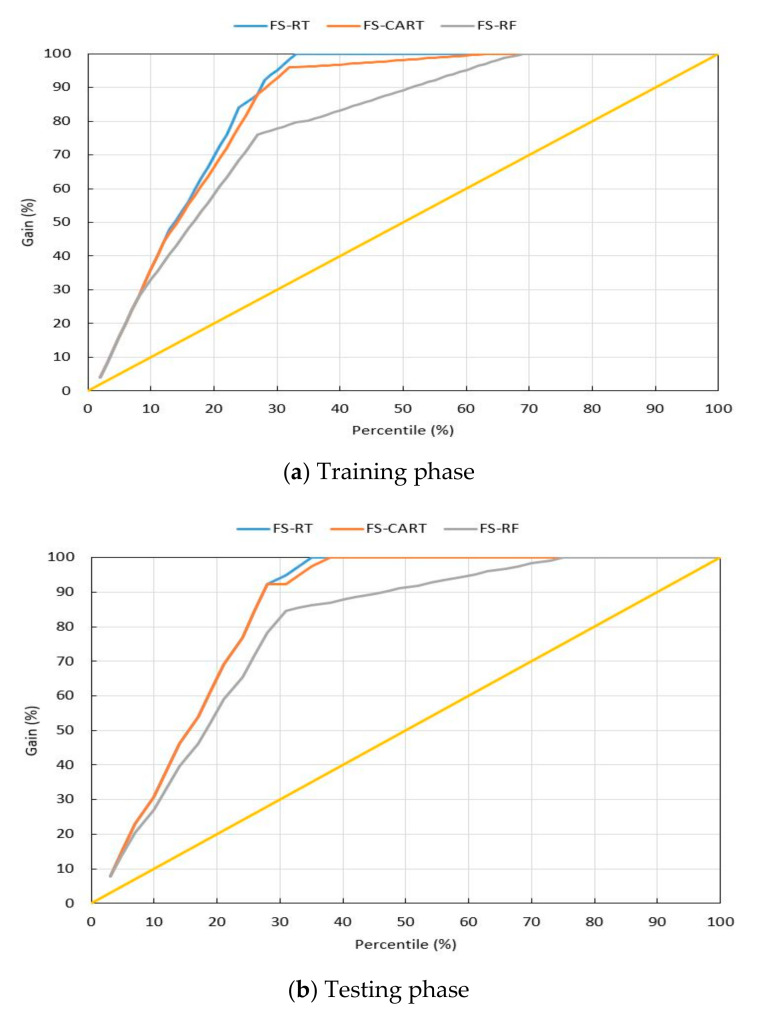
The gain chart of the hybrid models. (**a**) training phase, (**b**) testing phase.

**Figure 10:**
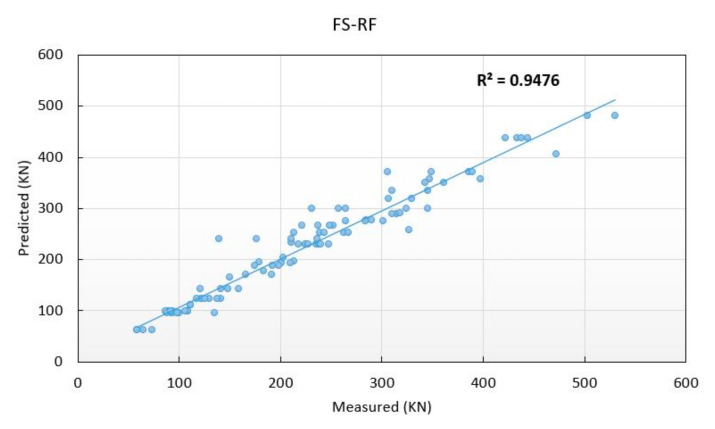

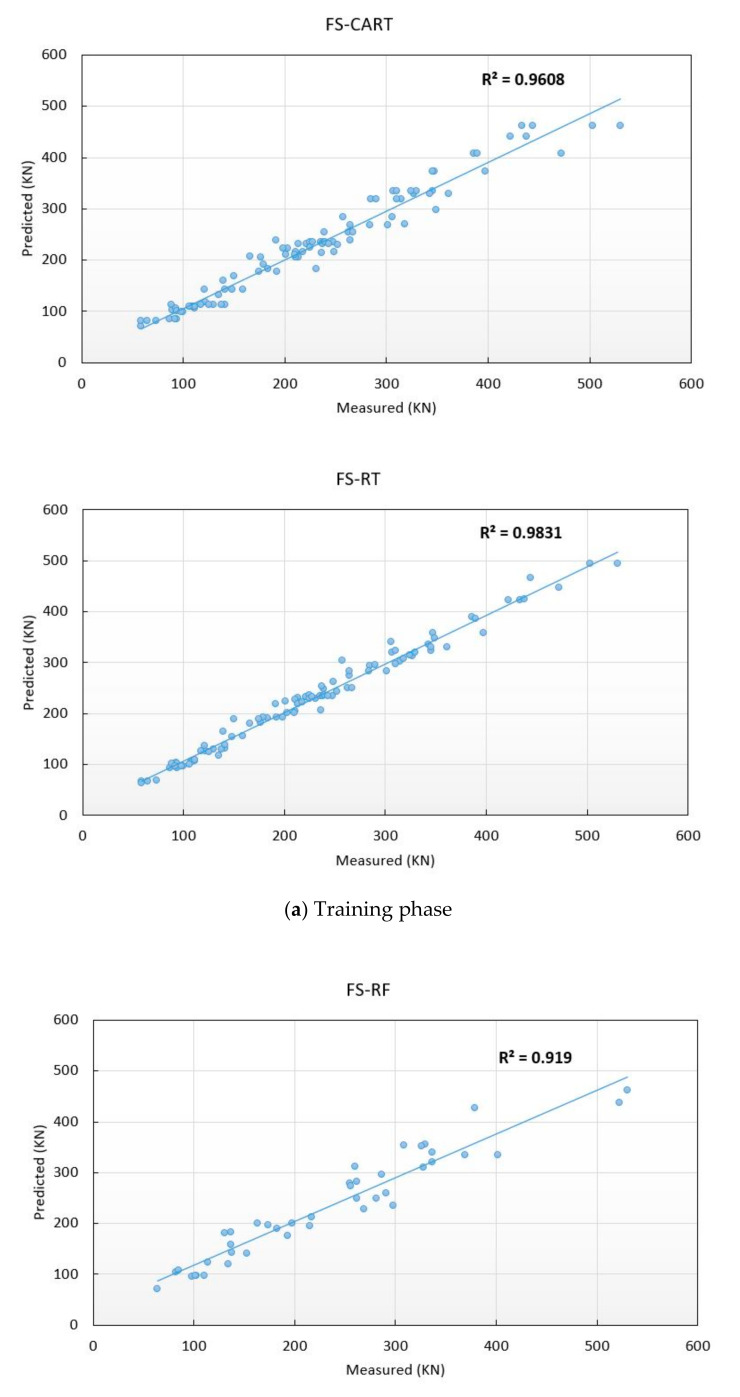

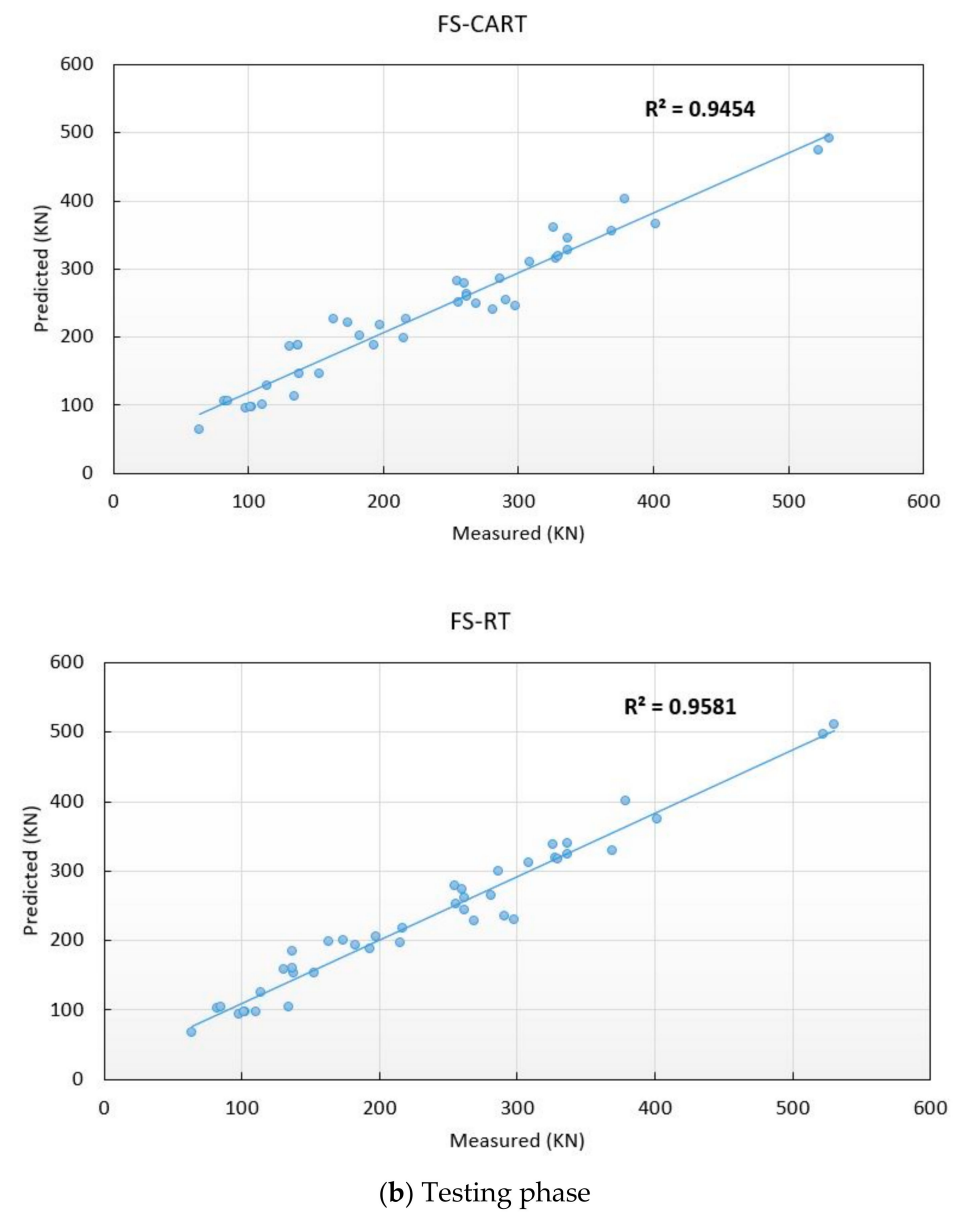
The correlations between measured and predicted punching shear capacity of the SFRC flat slabs by hybrid models. (**a**) training phase, (**b**) testing phase.

Since the criteria for comparing the models are important, the gain criterion was used for this purpose. Gain is a measure of the effectiveness of the models developed over the original data. This criterion shows how acceptable the performance of the models is. The more area the chart covers, the higher quality it offers. Figure 9 presents this category for the two sections of training and testing. Hybrid models generally have a more accurate prediction. However, the FS-RT model was found better than the other models in both stages of training and testing. In order to have a better understanding regarding the developed models, Figure 10 shows the correlations between measured and predicted punching shear capacity of the SFRC flat slabs by hybrid predictive models for both training and testing stages. Based on the results obtained in this study, it can be concluded that both single and hybrid ML techniques presented in this study are able to provide an acceptable accuracy level for the prediction of punching shear capacity of the SFRC flat slabs. However, if higher accuracy is of interest and necessary, the hybrid models can be utilized. The hybrid models are considered as powerful, practical, and easy to use models in estimating the punching shear capacity of SFRC flat slabs.

## 5. Conclusions

The current study introduced a number of ML techniques in order to estimate the punching shear capacity of SFRC flat slabs. The proposed techniques were applied to obtain an approximation of the mapping function between six descriptive variables (the depth of the slab, the effective depth of the slab, the length or radius of the column, the compressive strength of the concrete, the reinforcement ratio, and the fiber volume) and the output variable, i.e., the punching shear capacity. Moreover, the FS approach was implemented along with the tree models, i.e., RF, CART, and RT, for introducing new hybrid models. The results obtained from the methods indicated that the RT and CART models provided the best performance in predicting the punching shear capacity of the SFRC flat slabs. On the other hand, it was revealed that combining base models with FS could improve the accuracy of the results. Findings confirmed that the hybridized FS-RT method could be a promising tool for designing SFRC flat slabs. Future studies can evaluate other acceptance criteria for the breaking point as well as other sophisticated methods for preventing the overfitting of the FS-RT model. Moreover, the novel prediction models can be evaluated using an extended dataset, which can be based on the experiments carried out in recent studies. Finally, the proposed FS-based hybrid methods were shown applicable to solving other civil engineering problems. 

## Figures and Tables

**Table 1:** The statistical descriptions of the parameters to predict punching shear strength.

Parameters	Min	Max	STD	Average	Category
*d* (mm)	39	150	28.66	87.05	Input
*h* (mm)	55	180	33.22	110.82	Input
*bc* (mm)	60	225	46.33	131.96	Input
$\rho_{f}\;\left(\%\right)$	0	2	0.48	0.71	Input
$\rho \;\left(\%\right)$	0.37	2.53	0.51	0.99	Input
*f_c_* (Mpa)	14.2	108	21.29	41.65	Input
*V* (kN)	58.3	530	110.57	228.19	Output

**Table 2:** The results of various models in predicting the punching shear capacity of SFRC flat slabs.

Model	RF	CART	RT	FS-RF	FS-CART	FS-RT
Train Stage						
MAE	17.3402	15.7939	12.9320	17.1606	16.2317	10.9428
RMSE	29.5936	23.8149	19.5180	24.9310	21.5851	14.4965
R^2^	0.9295	0.9531	0.9695	0.9476	0.9608	0.9831
Test Stage						
MAE	27.3643	25.5189	22.2909	25.89257	21.1937	18.4813
RMSE	35.5025	32.3595	29.0696	32.8236	27.5289	23.8183
R^2^	0.9075	0.9282	0.9391	0.9190	0.9454	0.9581
